# OsbHLH062 regulates iron homeostasis by inhibiting iron deficiency responses in rice

**DOI:** 10.1007/s42994-025-00203-w

**Published:** 2025-03-03

**Authors:** Wujian Wang, Fengyu He, Hui Zhang, Yue Yang, Xiaojuan Wang, Yue Fu, Huixia Shou, Luqing Zheng

**Affiliations:** 1 College of Life Sciences, Nanjing Agricultural University, Nanjing, 210095 China; 2 State Key Laboratory of Plant Physiology and Biochemistry, College of Life Sciences, Zhejiang University, Hangzhou, 310058 China

**Keywords:** Fe homeostasis, OsbHLH062, Rice, TPL/TPRs, Fe homeostasis-related genes

## Abstract

Iron (Fe) homeostasis in plant cells is crucial for crop productivity and quality. An intricate transcriptional network involving numerous basic Helix-Loop-Helix (bHLH) transcription factors has been proposed to control Fe homeostasis. In the present study, we characterized rice (*Oryza sativa*) OsbHLH062, a member of the IVb subgroup of the bHLH family, demonstrating that it negatively regulates Fe-deficiency responses. OsbHLH062 represses transcription by recruiting TOPLESS/TOPLESS-RELATED co-repressors (TPL/TPRs) through its ethylene-responsive element binding factor-associated amphiphilic repression (EAR) motif. Under Fe deficiency, the expression of *OsbHLH062* is upregulated in roots and downregulated in shoots. Overexpression of *OsbHLH062* leads to decreased Fe accumulation in the shoot. Furthermore, OsbHLH062 interacts with POSITIVE REGULATOR OF IRON HOMEOSTASIS 1 (OsPRI1) and inhibits its transactivation activity, thereby negatively regulating the expression of many Fe homeostasis-related genes. These results indicate an important role for OsbHLH062 in regulating Fe homeostasis by negatively regulating Fe deficiency responses in rice. This knowledge will aid in the design of Fe-biofortified rice plants that can help to address the global issue of Fe deficiency.

## Introduction

Iron (Fe) is a cofactor for many enzymes and is involved in electron transfer reactions that play a crucial role in various key biological processes, including chlorophyll biosynthesis, photosynthesis, and respiration. Fe deficiency in plants leads to new leaf interveinal chlorosis and growth reduction (Broadley et al. 2012). Under alkaline conditions, Fe exists primarily as insoluble Fe (III) oxides and hydroxides, which limits it uptake by plants. Fe deficiency therefore becomes an important limiting factor for crop production and quality in alkaline soils, which account for one-third of cultivated soils worldwide (Briat et al. 2015). Excess Fe in cells, by contrast, results in toxicity via the Fenton reaction (Fenton 1894). In addition, people who rely primarily on plants for dietary Fe, especially women and children, are more likely to be affected by anemia related to Fe deficiency (Guerinot 2001; Hanikenne et al. 2021). Therefore, it is essential to understand the molecular mechanism(s) by which plants strictly regulate Fe homeostasis.

Plants have evolved two strategies to obtain enough Fe from soils (Römheld 1987). Strategy I is used by nongraminaceous plants. Strategy II, also called the chelation strategy, is used by graminaceous plants and relies primarily on the biosynthesis and secretion of mugineic acid (MA) family phytosiderophores into the rhizosphere (Römheld 1987). These phytosiderophores are nonprotein amino acids that can solubilize and chelate Fe (III), forming an Fe (III)-MA complex that can be taken up into roots by yellow stripe 1 and yellow stripe 1-like (YS1 and YSL) transporters (Kobayashi and Nishizawa 2012). In rice (*Oryza sativa* L.), three key enzymes—nicotianamine synthase (NAS), nicotianamine aminotransferase 1 (NAAT1), and deoxymugineic acid synthase 1 (DMAS1)—mediate the biosynthesis of MAs (Bashir et al. 2006, 2017; Cheng et al. 2007; Inoue et al. 2003, 2008). Rice TRANSPORTER OF MAS 1 (OsTOM1) is responsible for the secretion of the MAs, and OsYSL15 mediates the uptake of the Fe (III)-MA complex (Inoue et al. 2009; Nozoye et al. 2011). Rice plants can also absorb the more soluble Fe (II) directly via IRON REGULATED TRANSPORTER 1 (OsIRT1) (Ishimaru et al. 2006).

The expression of genes involved in Fe uptake via Strategy II is induced in response to low Fe availability. In rice, *Orzya sativa* FER-LIKE Fe DEFICIENCY-INDUCED TRANSCRIPTION FACTOR/BASIC HELIX LOOP HELIX 156 (OsFIT/OsbHLH156) can interact with the iron-related bHLH transcription factor (TF) (OsIRO2) and promote the nuclear accumulation of OsIRO2 to directly regulate the expression of *OsNAS1*, *OsNAS2*, *OsNAAT1*, *OsDMAS1*, *OsTOM1*, and *OsYSL15*. Both *OsFIT*/*OsbHLH156* and *OsIRO2* are induced by Fe deficiency (Liang et al. 2020; Ogo et al. 2007; Wang et al. 2020b). The induction of *OsIRO2* is strongly controlled by POSITIVE REGULATOR OF IRON HOMEOSTASIS 1 (OsPRI1/OsbHLH060), OsPRI2 (OsbHLH058), OsPRI3 (OsbHLH059), and OsPRI4 (bHLH057), all of which belong to the IVc clade of bHLH TFs (Kobayashi et al. 2019; Wang et al. 2022a; Zhang et al. 2017, 2020).

OsIRO3, a member of the IVb bHLH TF clade, is a negative regulator of Fe-deficiency response signaling (Wang et al. 2020a, c; Zheng et al. 2010). It acts by directly or indirectly binding to OsPRI1/2 and repressing the expression of *OsIRO2* (Li et al. 2022a). Similarly, OsbHLH061, another clade IVb bHLH TF, is also an interacting partner of OsPRI1 that inhibits its transcriptional activity (Wang et al. 2022b). Both OsbHLH061 and OsIRO3 can recruit TOPLESS/TOPLESS-RELATED (OsTPL/TPR) co-repressors through their ethylene-responsive element binding factor-associated amphiphilic repression (EAR) motifs, which are required for their transcriptional repression activity (Li et al. 2022a; Wang et al. 2022b). Knocking out *OsbHLH061* increases the expression of many Fe homeostasis-related genes and Fe accumulation in the shoot under Fe-sufficient conditions. More Fe is accumulated in shoots of the *iro3* loss-of-function mutant than in those of the wild type under Fe-deficient conditions. Notably, the new leaves of *iro3* mutants show brown necrotic lesions in response to Fe deficiency (Carey-Fung et al. 2022; Li et al. 2022a; Wang et al. 2020a, c, 2022b). This might be due to the opposite transcriptional responses of *OsIRO3* and *OsbHLH061* to Fe deficiency, with *OsIRO3* being induced and *OsbHLH061* being repressed (Wang et al. 2020c, 2022b; Zheng et al. 2010).

These results demonstrate that although OsIRO3 and OsbHLH061 are paralogous and have similar biochemical functions, they play different roles in modulating Fe homeostasis and the Fe-deficiency response in rice. OsbHLH062 and OsbHLH064 are two additional members of the clade IVb bHLH TFs. Phylogenetic analysis reveals that OsbHLH062 is more closely related to OsbHLH061 and OsIRO3 than it is to OsbHLH064 (Li et al. 2006; Zheng et al. 2010). Because the function of OsbHLH062 has not yet been characterized, we aimed to clarify whether and how OsbHLH062 regulates Fe homeostasis in rice.

Here, we show that OsbHLH062 is a transcriptional repressor and that its transcriptional repression activity is dependent on its EAR motif, which can recruit TPL/TPR co-repressors. In response to Fe deficiency, the expression of *OsbHLH062* is upregulated in roots, but downregulated in shoots. Genetic analysis suggested that OsbHLH062 functions as a negative regulator of Fe deficiency response signaling. Furthermore, we confirmed that OsbHLH062 interacts with OsPRI1 and represses its transactivation activity.

## Results

### OsbHLH062 is a transcription repressor and interacts with OsTPL/TPRs

**Fig. 1:**
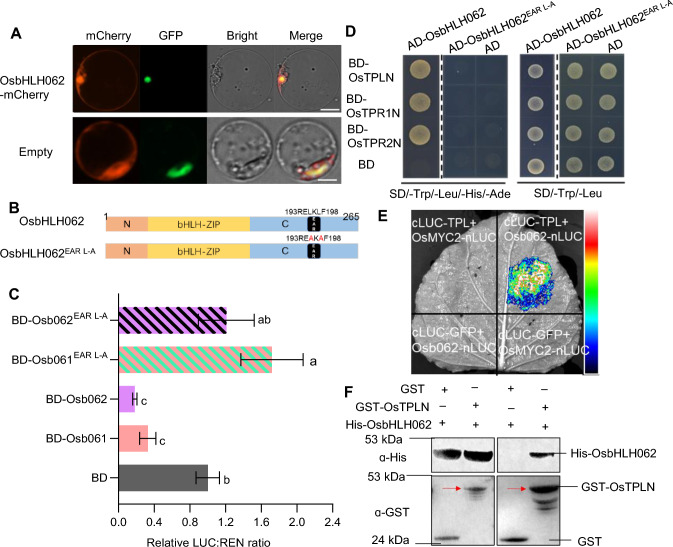
OsbHLH062 is a transcriptional repressor. **A** Subcellular localization of OsbHLH062 in rice protoplasts. OsbHLH062 was fused with mCherry to create the fusion protein OsbHLH062-mCherry. A nuclear protein OsAPI5, was fused with GFP as a nuclear marker. Scar bar, 10 µM. **B** Schematic diagrams of OsbHLH062 and its mutation in the EAR motif (OsbHLH062^EAR L−A^). **C** Transcriptional activity testing of OsbHLH062 and OsbHLH062^EAR L−A^ using dual-luciferase expression assays. OsbHLH061 and OsbHLH061^EAR L−A^ served as positive controls. The firefly luciferase to renilla luciferase (LUC: REN) ratios indicate transcriptional activities, with the LUC to REN ratio in the BD empty control set to 1. Data are represented as means ± SD (*n* = 3). Different letters above each bar indicate statistically significant differences (*P* < 0.05) determined by one-way ANOVA followed by Tukey’s multiple comparison test. **D** Yeast-two-hybrid assay. The coding sequences of OsbHLH062 and OsbHLH062^EAR L−A^ were fused with the activation domain (AD), while the N-terminal coding sequences of OsTPL/TPRs were fused with the binding domain (BD). Yeasts co-transformed with different BD and AD vector combinations were plated on SD/-Trp/-Leu and SD/-Trp/-Leu/-His/-Ade media. Interaction was indicated by the ability of cells to grow on SD/-Trp/-Leu/-His/-Ade medium. **E** Split luciferase complementation assays. OsbHLH062 and OsTPL were fused with the N-terminal and C-terminal fragments of luciferase (LUC). Proteins shown in the figure were co-expressed in *Nicotiana benthamiana* leaves, and LUC reconstitution was used to check interactions among the different combinations. **F** Pull-down assay to detect interaction between OsbHLH062 and OsTPL. The N-terminal part of OsTPL was fused with the GST tag, while OsbHLH062 was fused with the His tag. His-OsbHLH062 was incubated with GST-OsTPLN or GST and pulled down using glutathione-agarose beads. Proteins were analyzed by immunoblotting with the anti-His or anti-GST antibodies

To investigate whether OsbHLH062 is localized in the nucleus, where it might regulate transcription, the construct *CaMV35S*_*pro*_*:OsbHLH062-mCherry* was generated and transiently expressed in rice protoplasts, using *CaMV35S*_*pro*_*:mCherry* as a negative control and *CaMV35S*_*pro*_*:OsAPI5-GFP* as a marker for the nucleus. We observed that the fluorescence signal of OsbHLH062-mCherry overlapped with that of OsAPI5-GFP and was also present in the cytoplasm (Fig. 1A). This result demonstrated that OsbHLH062 is localized to both the nucleus and cytoplasm.

An EAR-like motif, 193-RELKLF-198, was identified in the C-terminal region of OsbHLH062 (Fig. 1B), as well as in OsbHLH061 and OsIRO3 (Li et al. 2022a; Wang et al. 2022b). TPL family proteins can be recruited by proteins containing the EAR motif, producing a transcription-repressing complex (Kagale and Rozwadowski 2011). To test for OsbHLH062 transcriptional repression activity, a dual luciferase (LUC) assay was performed. LUC under the control of a mini cauliflower mosaic virus 35S promoter with five repeats of the GAL4 TF binding element was used as the reporter, and Renilla luciferase (REN) under the control of the *35S* promoter was used as an internal control. The *CaMV35S*_*pro*_*:BD*, *CaMV35S*_*pro*_*:-OsbHLH061/062*, and *CaMV35S*_*pro*_*:BD-OsbHLH061/062*^*EAR L−A*^ were used as effectors (Fig. S1). As with the positive control vectors *CaMV35S*_*pro*_*:BD-OsbHLH061* and *CaMV35S*_*pro*_*:BD-OsbHLH061*^*EAR L−A*^, expression of *CaMV35S*_*pro*_*:BD-OsbHLH062* significantly decreased the LUC/REN value. This repression was not observed upon expression of *CaMV35S*_*pro*_*:BD-OsbHLH062*^*EAR L−A*^ (Fig. 1C). These results indicated, that OsbHLH062 has transcriptional repression activity.

We further examined whether the EAR motif in OsbHLH062 is required for recruiting TPL family proteins. The N-terminal regions of three rice TPL/TPR proteins (OsTPR1N, OsTPR2N, and OsTPLN) are reported to be necessary for interaction with the EAR motif (Ke et al. 2015). In a yeast two-hybrid (Y2H) assay, we determined that these regions bind to OsbHLH062 (Fig. 1D). Moreover, we demonstrated the interaction of OsbHLH062 with OsTPL, *in planta,* using a split-LUC complementation image assay (LCI; Fig. 1E). Finally, a pull-down assay was used to confirm the interaction between OsbHLH062 and OsTPL in vitro. This showed that GST-TPLN, but not the GST negative control, could pull down His-OsbHLH062 (Fig. 1F). To further examine whether the EAR motif is required for OsbHLH062 binding to OsTPL/TPRs, we generated a version of OsbHLH062 that was mutated in the EAR motif (Fig. 1B). This mutation completely abolished the interaction between OsbHLH062 and OsTPL/TPRs (Fig. 1D). In summary, these data show that OsbHLH062 directly interacts with OsTPL/TPRs via its EAR motif, and that the EAR motif is essential for its transcriptional repression activity.

### Expression pattern of *OsbHLH062*

**Fig. 2:**
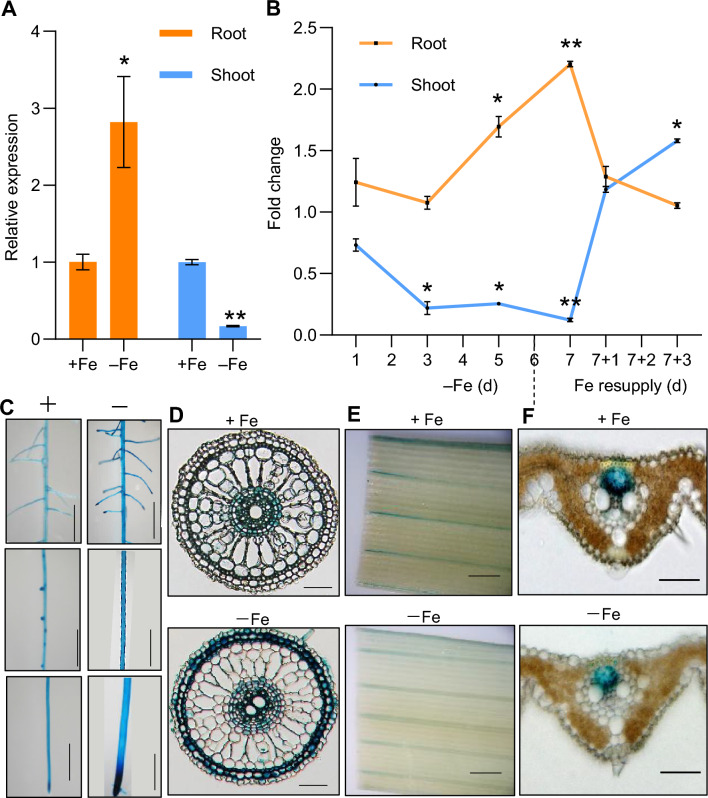
Fe-dependent expression pattern of *OsbHLH062.* **A** Two-week-old wild type (WT) plants were transferred to Fe-deficient conditions (–Fe) for 8 d. Roots and shoots were sampled for gene expression analysis. **B** WT plants were subjected to Fe-deficient conditions (–Fe) for 1, 3, 5, 7 d, after 7 d the plants were shifted back to normal conditions for additional 1 or 3 d. Data are presented as means ± SD of three biological replicates. Asterisks indicate significant differences between normal and –Fe or Fe resupply conditions based on Student’s *t* test (*, *P* < 0.05; **, *P* < 0.01). **C-F** Histochemical staining of GUS activity in *OsbHLH062*_*Pro*_:*GUS* transgenic rice plants.** C** Different root zones under Fe-sufficient and -deficient conditions. **D** Transverse section of roots. **E** Leaf blades under Fe-sufficient and -deficient conditions. **F** Transverse section of leaf blade. Bars = 0.6 cm in (C and E) and 60 µm in (D and F). ‘ + ’ indicates normal conditions; ‘ – ’ indicates Fe-deficient conditions

We next analyzed the expression of *OsbHLH062* in response to Fe deficiency. Fourteen-day-old rice plants were exposed to Fe deficiency for 8 days, after which roots and shoots were collected for RNA extraction. The results of reverse-transcription quantitative PCR (RT-qPCR) showed that *OsbHLH062* is induced by Fe deficiency in roots but is repressed in shoots (Fig. 2A). To further analyze *OsbHLH062* expression, we performed a time-course Fe-deficiency experiment, by exposing rice seedlings to Fe deficiency for 1 week, and subsequently resupplying Fe for 3 days. In roots, *OsbHLH062* transcript abundance increased on day 5 and further increased by day 7 of Fe deficiency, but quickly decreased upon Fe resupply (Fig. 2B). By contrast, in shoots, *OsbHLH062* was downregulated by day 3 under Fe-deficient conditions. Following Fe resupply, we noted a rapid increase in *OsbHLH062* transcript abundance in shoots after 1 day, reaching a peak on day 3 (Fig. 2B).

To examine the tissue-dependent expression of *OsbHLH062*, the 2,137-bp promoter of *OsbHLH062* was fused upstream of the sequence encoding beta-glucuronidase (GUS). GUS staining results suggested that the expression of *OsbHLH062-GUS* was induced by Fe throughout the root, and it was higher in root tips than in other parts of the root (Fig. 2C). In leaves, *OsbHLH062* was highly expressed in the veins (Fig. 2E). Furthermore, histochemical staining of root and leaf cross-sections showed higher expression of *OsbHLH062* in the stele and exodermis of the roots, as well as in the phloem of the leaves (Fig. 2D, F).

### OsbHLH062 negatively regulates Fe deficiency responses

To evaluate the role of OsbHLH062 in the regulation of Fe homeostasis in rice, we created two independent loss-of-function mutations of *OsbHLH062*. Two 20-bp sequences in the first exon of *OsbHLH062* were selected for guide RNA (Fig. S2A). Two homozygous mutants, *osbhlh062-1* and *osbhlh062-2*, were identified by Sanger sequencing. These mutants contained an insertion of ‘A’ in the gRNA1 or gRNA2 site, respectively (Fig. S2B), resulting in a frameshift mutation that caused a premature stop codon (Fig. S2C). We then compared the growth of the wild type (WT) and the *osbhlh062* mutant plants under both normal (2 µM FeSO_4_) and Fe-limited (0 µM FeSO_4_) conditions. After 8 days of Fe deficiency, growth of the *osbhlh062* mutants was not significantly different from that of the WT. This was reflected by the Soil and Plant Analysis Development (SPAD) value, root length, and shoot height, which were similar in the WT and *osbhlh062* mutants under Fe-sufficient and Fe-deficient conditions (Fig. S3A–D).

Furthermore, we measured Fe concentrations in the shoots and roots of the WT and *osbhlh062* mutant plants using inductively coupled plasma mass spectrometry (ICP-MS). The Fe concentration in shoots and roots of the *osbhlh062* mutants was not significantly different from that in the WT, regardless of external Fe availability (Fig. S3E, F). No obvious difference in response to Fe deficiency was observed in the *osbhlh062* mutants, compared with the WT, possibly due to the redundancy of OsbHLH062 and its paralogs OsbHLH061 and OsIRO3.

**Fig. 3:**
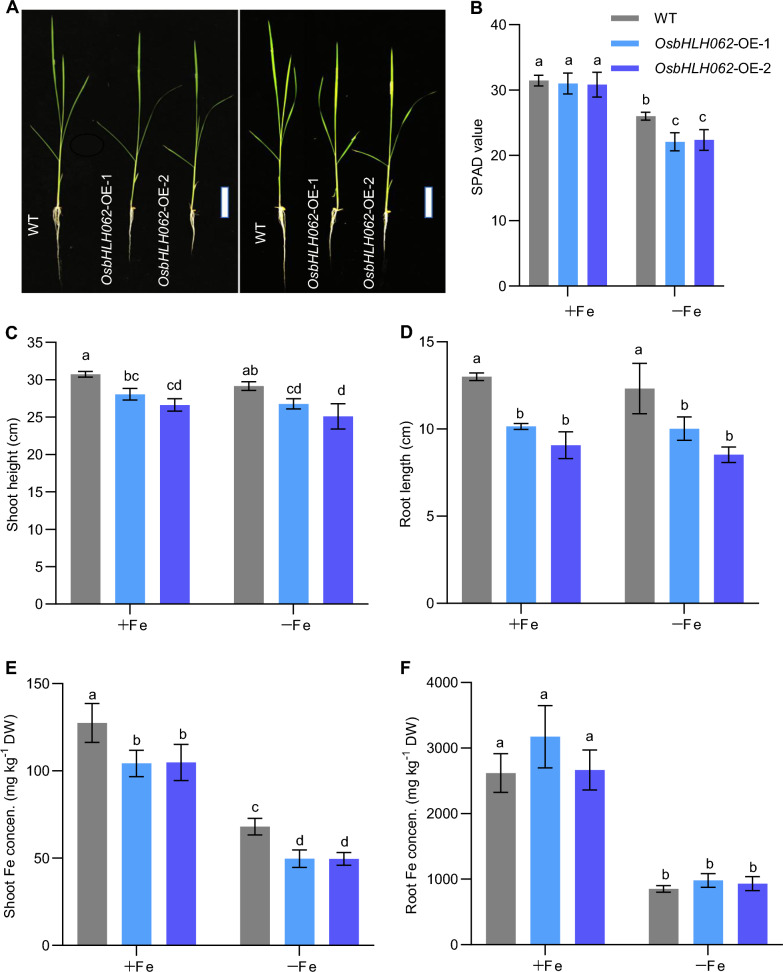
Phenotypic analysis of *OsbHLH062* overexpression lines. Fourteen-day-old seedlings were transferred to a solution containing 2 μM FeSO_4_ (+ Fe) or without FeSO_4_ (–Fe) for 8 d. **A** Images of wild-type (WT) and *OsbHLH062* overexpression lines. Scale bars = 5 cm. **B** SPAD value of new leaves in WT and *OsbHLH062* overexpression lines. **C** Shoot heights of WT and *OsbHLH062* overexpression lines. **D** Root lengths of WT and *OsbHLH062* overexpression lines. **E** Shoot Fe concentrations in WT and *OsbHLH062* overexpression lines. **F** Root Fe concentrations in WT and *OsbHLH062* overexpression lines. Data in panels B-D represent means ± SD from four biological replicates, while panels E and F present means ± SD from three biological replicates. Statistical significance was determined using one-way ANOVA followed by Tukey’s multiple comparison test (*P* < 0.05). Means with different letters indicate statistically significant differences

To further clarify the function of OsbHLH062 in the regulation of Fe homeostasis, we generated two independent lines of *OsbHLH062-*overexpressing rice plants harboring the full-length coding sequence (CDS) of *OsbHLH062* driven by the *35S* promoter (Os*bHLH062*-OE-1 and Os*bHLH062*-OE-2). The expression levels of *OsbHLH062* in Os*bHLH062*-OE-1 and Os*bHLH062*-OE-2 plants were about 5.6 and 6.3 times the levels in WT plants, respectively (Fig. S4). In comparison with the WT, both *OsbHLH062* overexpression lines displayed chlorosis of new fully expanded leaves, and this was associated with lower SPAD values under Fe-deficient conditions (Fig. 3A, B). Under both Fe-sufficient and Fe-deficient conditions, the root lengths and shoot heights of *OsbHLH062*-overexpressing plants were significantly lower than those of the WT (Fig. 3C, D). Moreover, overexpression of *OsbHLH062* led to significantly lower Fe accumulation in shoots, being 18% lower (Fe-sufficient) and 28% lower (Fe-deficient) than that in the WT (Fig. 3E). In roots, the Fe concentration was not significantly different between the WT and *OsbHLH062*-overexpressing plants (Fig. 3F). Collectively, these data suggest that OsbHLH062 negatively regulates Fe accumulation in shoots.

We further investigated the growth of *osbhlh062* mutants in response to excess Fe, using *osbhlh061* mutants, which are sensitive to excess Fe (Wang et al. 2022b), as a positive control. The relative shoot dry weight of seedlings grown under 200 µM FeSO_4_ compared with the control condition (2 µM FeSO_4_) showed that the performance of *osbhlh062* mutants was similar to that of the WT, and that growth of *osbhlh061* mutants was more sensitive to excess Fe than was that of the WT (Fig. S5A, B). Consistent with these phenotypes, *osbhlh061* accumulated more Fe in shoots than the WT, whereas *osbhlh062* had a similar Fe concentration in the shoot (Fig. S5C).

### Expression of Fe homeostasis-related genes is repressed by OsbHLH062

**Fig. 4:**
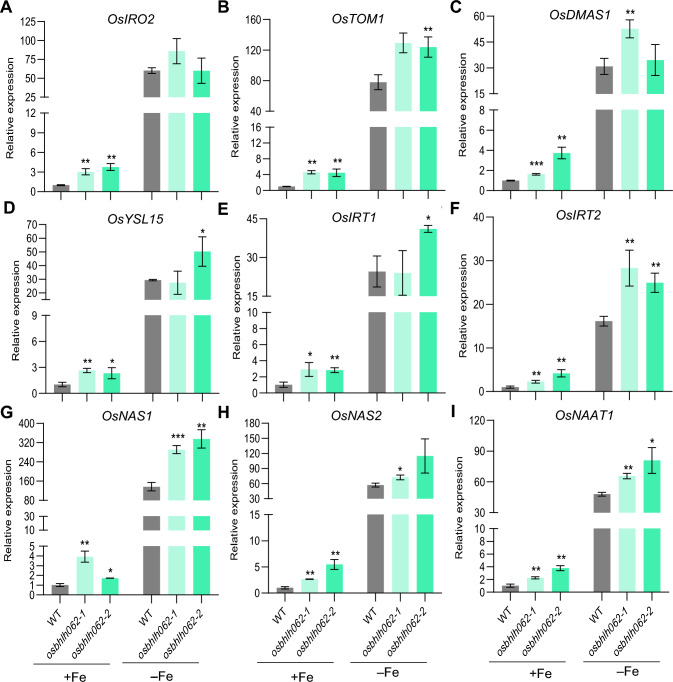
Expression of Fe homeostasis-related genes in WT and *osbhlh062* mutants. Fourteen-day-old rice seedlings of WT, *osbhlh062-1*, and *osbhlh062-2* were transferred to Fe-sufficient or Fe-deficient conditions and grown for 4 d. Roots were sampled for RNA extraction and gene expression analysis. Relative expression levels of *OsIRO2* (**A**), *OsTOM1* (**B**), *OsDMAS1* (**C**), *OsYSL15* (**D**), *OsIRT1* (**E**), *OsIRT2* (**F**), *OsNAS1* (**G**), *OsNAS2* (**H**), and *OsNAAT1* (**I**) was quantified by qRT-PCR. With *OsActin1* serving as the internal standard. The expression levels in WT under Fe-sufficient conditions were normalized to 1. Data are presented as means ± SD from three biological replicates. Asterisks indicate statistically significant differences between WT and *osbhlh062* mutants, determined by two-tailed Student’ s *t-*test (*, *P* < 0.05; **, *P* < 0.01)

**Fig. 5:**
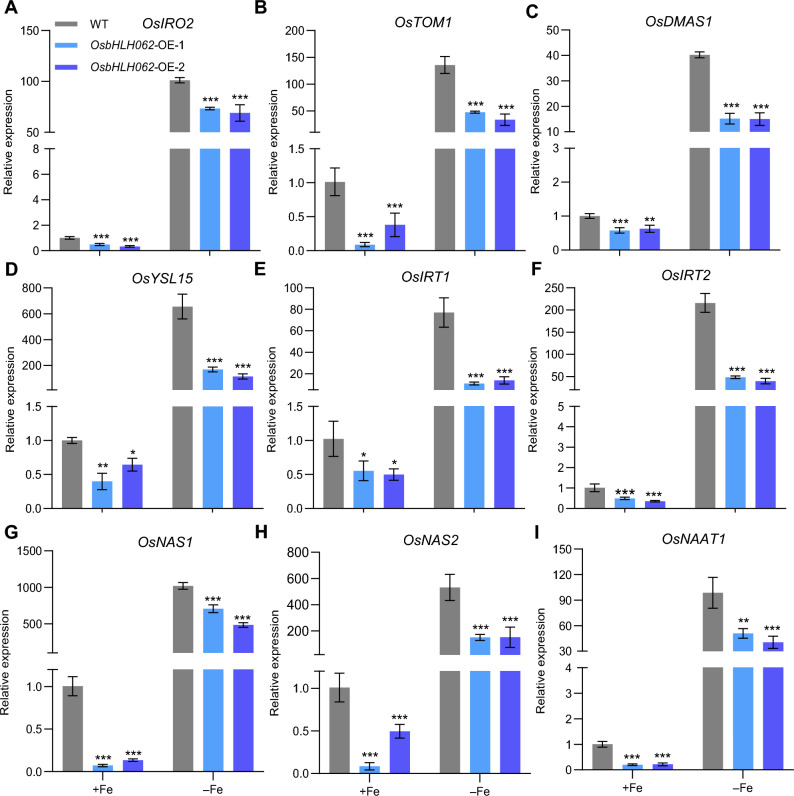
Expression of Fe deficiency-responsive genes in WT and *OsbHLH062* overexpression lines. Fourteen-day-old rice seedlings were grown in Fe-sufficient (+Fe) and Fe-deficient (–Fe) conditions for 4 d. Roots were harvest for gene expression analysis. Relative expression levels of *OsIRO2* (**A**), *OsTOM1* (**B**), *OsDMAS1* (**C**), *OsYSL15* (**D**), *OsIRT1* (**E**), *OsIRT2* (**F**), *OsNAS1* (**G**), *OsNAS2* (**H**), and *OsNAAT1* (**I**) were determined by qRT-PCR. With *OsActin1* serving as internal control. Gene expression levels in WT under + Fe conditions were normalized to 1. Values are presented as means ± SD from three biological replicates. Asterisks indicate significant differences between WT and *OsbHLH061/062/063* overexpression lines as determined by two-tailed Student’ s *t-*test (*, *P* < 0.05; **, *P* < 0.01; ***, *P* < 0.001)

To investigate the role of OsbHLH062 in regulating the transcription of genes responsive to Fe deficiency, we examined the expression of these genes in the WT and *osbhlh062* mutants under both Fe-sufficient and Fe-deficient conditions. We observed that marker genes for the Fe-deficiency response—*OsIRO2*, *OsTOM1*, *OsDMAS1*, *OsYSL15*, *OsIRT1*, *OsIRT2*, *OsNAS1*, *OsNAS2*, and *OsNAAT1*—were upregulated in the *osbhlh062* mutants under normal conditions. Under Fe-deficient conditions, the expression levels of *OsTOM1*, *OsIRT2*, *OsNAS1*, and *OsNAAT1* were still higher in the *osbhlh062* mutants compared with their levels in the WT (Fig. 4). We therefore investigated differences in the expression of these genes in *OsbHLH062*-overexpressing transgenic plants. Compared with the WT, the expression levels of these genes were largely downregulated in plants overexpressing *OsbHLH062,* under both Fe-sufficient and Fe-deficient conditions (Fig. 5). In addition, the expression levels of *OsbHLH061* and *OsIRO3* in the *osbhlh062* mutants under Fe-sufficient conditions were similar to levels in the WT (Fig. S6). Together, the results of gene expression analysis indicated that OsbHLH062 is a negative regulator of the expression of several Fe-homeostasis-related genes.

### OsbHLH062 interacts with OsPRI1 and inhibits its transactivation activity

**Fig. 6:**
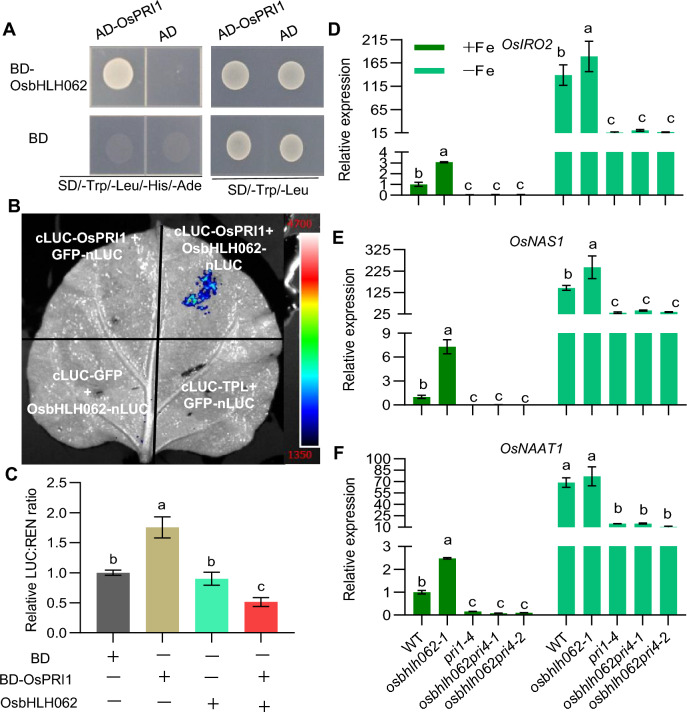
Interaction of OsbHLH062 with OsPRI1 and its effect on transactivation. **A** Interaction of OsbHLH062 with OsPRI1 was assessed using a yeast-two-hybrid (Y2H) assay. The coding sequences (CDS) of OsbHLH062 and OsPRI1 were cloned into the *pGBKT7* and *pGADT7* vectors, respectively. Yeast co-transformants were plated on SD/-Trp/-Leu for viability and SD/-Trp/-Leu/-His/-Ade to assess interaction. **B** LCI assay demonstrating the interaction of OsbHLH062 with OsPRI1. Various combinations of nLUC and cLUC fusion proteins are indicated, LUC reconstitution was employed to test interactions, with GFP used as a negative control. **C** OsbHLH062 suppresses the transactivation of OsPRI1. The ratio of LUC to REN activity reflects promoter activity, with the LUC:REN value for the empty vector set to 1. **D**–**F** Gene expression analysis of *OsIRO2* (D), *OsNAS1* (**E**), and *OsNAAT1* (**F**) in 14-d-old rice seedlings of WT, *osbhlh062-1*, *pri1-4*, *osbhlh062pri1-1*, and *osbhlh062pri1-2* grown in +Fe and –Fe for 4 d. Relative expression was determined by RT-qPCR, using *OsActin1* as internal control; gene expression levels in WT under + Fe were set to 1. Values are means ± SD of three biological replicates. Means with different letters indicate statistically significant differences as determined by one-way ANOVA followed by Tukey’s multiple comparison test (*P* < 0.05)

To test whether OsbHLH062, OsbHLH061, and OsIRO3 (Li et al. 2022a; Wang et al. 2022b), interacts with OsPRI1, a master regulator of Fe homeostasis, we performed Y2H and LCI assays. OsbHLH062 fused to the GAL4 DNA-binding domain (BD) was used as the bait, and OsPRI1 fused to the GAL4 activating domain (AD) was used as prey. We observed a direct interaction between OsbHLH062 and OsRPI1 (Fig. 6A). The interaction between OsbHLH062 and OsPRI1 was confirmed *in planta* using a LCI assay (Fig. 6B). Finally, interaction of OsPRI1 with OsbHLH062 in vitro was demonstrated using a pull-down assay (Fig. S7).

Furthermore, we determined that OsbHLH062, as well as OsIRO3, interacts with other proteins (OsPRI2, 3, and 4) (Li et al. 2022a) in vivo in Y2H and LCI assays (Fig. S8). The physical interaction between OsbHLH062 and OsPRIs led us to investigate whether OsbHLH062 affects the transcriptional activity of OsPRIs, using OsPRI1 as a representative OsPRI protein. To determine whether OsbHLH062 directly represses transactivation of OsPRI1 via a protein–protein interaction, a GAL4-based dual LUC assay was performed. In this assay, *GAL4*_*pro*_*:LUC* and *35S:REN* constructs were used as a reporter and an internal control, respectively (Fig. S9). Expression of the effector plasmid p35S:BD-OsPRI1, but not p35S:OsbHLH062, significantly increased LUC reporter activity. By contrast, co-expression of OsbHLH062 with OsPRI1 reduced the relative reporter activity compared with expression of the empty vector p35S::BD (Fig. 6C). These results show that OsbHLH062 can repress downstream gene expression by inhibiting the transactivation of OsPRI1.

To further clarify the genetic interaction between *OsbHLH062* and *OsPRI1*, *pri1osbhlh062-1* and *pri1osbhlh062-2* double mutants were generated using the CRISPR-Cas9 system. For *OsPRI1*, there was an insertion of ‘A’ in both the *pri1osbhlh062-1* and the *pri1osbhlh062-2* double mutants. For *OsbHLH062*, there was a deletion of ‘G’ in *pri1osbhlh062-1* and an insertion of ‘TG’ in *pri1 osbhlh062-2* (Fig. S10). *OsIRO2*, *OsNAS1*, and *OsNAAT1* were selected as representative downstream genes for expression analysis in the WT and *osbhlh062-1*, *pri1-4*, *pri1osbhlh062-1*, and *pri1osbhlh062-2* plants grown under Fe-sufficient and Fe-deficient conditions. Under Fe-sufficient conditions, the transcript abundances of *OsIRO2*, *OsNAS1*, and *OsNAAT1* decreased in the *pri1* mutant in comparison with the WT (Wang et al. 2022b; Zhang et al. 2017), but they increased in the *osbhlh062* mutant (Fig. 6D–F). The expression of *OsIRO2*, *OsNAS1*, and *OsNAAT1* was significantly lower in *pri1osbhlh062-1* and *pri1osbhlh062-2* than in the WT or *osbhlh062-1* under both Fe-sufficient and Fe-deficient conditions, but it was similar to expression in the *pri1* mutant (Fig. 6D–F). These results suggest that OsbHLH062 is genetically upstream of OsPRI1.

## Discussion

Plants can sense Fe deficiency or Fe excess and transmit the appropriate signals to TFs that tightly regulate the transcription of genes involved in Fe uptake, transport, and distribution (Riaz and Guerinot 2021; Gao and Dubos 2021). In rice, bHLH clade IVc TFs and two bHLH clade IVb TFs (OsIRO3 and OsbHLH061) are positioned upstream in the Fe homeostasis network and they regulate the expression of Fe homeostasis-related genes, including *OsIRO2* (Li et al. 2022a; Wang et al. 2022b). In the present study, we confirmed that OsbHLH062, another clade IVb TF, plays a critical role in maintaining Fe homeostasis in rice.

OsbHLH061, OsbHLH062, and OsIRO3 all possess an EAR or EAR-like motif in their C-terminal regions (Fig. 1B) (Wang et al. 2022b). Previous studies, including our own, have shown that OsbHLH061 and OsIRO3 can recruit OsTPL/TPR co-repressors through their EAR motifs, thereby conferring their transcriptional repression activity (Li et al. 2022a; Wang et al. 2022b). Here, we demonstrated that OsbHLH062 is located in the nucleus and exhibits transcriptional repression activity. Additionally, we determined that OsbHLH062 directly interacts with OsTPL/TPRs via its EAR motif, which is crucial for its transcriptional repression activity (Fig. 1). These findings suggest that the three IVb TFs (OsbHLH061, OsbHLH062, and OsIRO3) share a similar biochemical function as transcriptional repressors.

Transient expression of OsbHLH062-mCherry in rice protoplasts revealed its presence in both the nucleus and cytoplasm. Additionally, OsbHLH061 was also shown to localize to both compartments (data not shown). Furthermore, three members of the *Arabidopsis thaliana* (Arabidopsis) bHLH IVb subgroup (AtbHLH11, AtbHLH121, and AtPYE) are similarly localized in both the nucleus and the cytoplasm (Lei et al. 2020; Li et al. 2022b; Pu and Liang 2023). This suggests that bHLH IVb proteins are likely conserved in their dual localization. In *Arabidopsis*, bHLH IVc proteins can facilitate the nuclear import of AtbHLH11, AtbHLH121, and AtPYE, thereby precisely regulating the expression of Fe uptake genes and maintaining Fe homeostasis (Lei et al. 2020; Li et al. 2022b; Pu and Liang 2023). Generally, the shuttling of proteins between the cytoplasm and nucleus serves as a rapid response mechanism that allows plants to adapt to environmental fluctuations. In rice, OsbHLH156/OsFIT promotes the nuclear accumulation of OsIRO2 from the cytoplasm, quickly enhancing the expression of Fe uptake genes to adjust to low Fe availability (Liang et al. 2020; Wang et al. 2020b). These findings suggest that dynamic localization changes between the nucleus and cytoplasm for certain regulators of Fe homeostasis are critical for maintaining Fe balance in plants. Additionally, we determined that OsPRIs physically interact with OsbHLH062 (Fig. 6, Figs. S7, S8) and OsbHLH061 (Wang et al. 2022b). Therefore, it is essential to examine whether OsPRIs enhance the nuclear localization of OsbHLH062 and/or OsbHLH061, further emphasizing the role of conditional nuclear accumulation in regulating plant Fe homeostasis.

We investigated the functions of OsbHLH062 in regulating Fe homeostasis in rice by using inactivation mutants and overexpression lines of *OsbHLH062* (Figs. S2 and S4). Analysis of *osbhlh062* loss-of-function mutants revealed that under both Fe-sufficient and Fe-deficient conditions, neither growth performance nor Fe concentration in shoots and roots was significantly affected by knocking out *OsbHLH062* (Fig. S3). As anticipated, many Fe-deficiency-responsive genes were upregulated in the *osbhlh062* mutants under Fe-sufficient conditions, and some remained upregulated under Fe-deficient conditions (Fig. 4). These findings suggest that OsbHLH062 functions redundantly with OsbHLH061 and/or OsIRO3. Indeed, several Fe-deficiency genes were upregulated in the *iro3* mutants under Fe-sufficient conditions, whereas Fe concentrations were similar to those of the WT under the same conditions (Li et al. 2022a; Wang et al. 2020a, 2020c), mirroring the results observed in *osbhlh062* mutants (Fig. S3 and Fig. 4).

Therefore, we propose that OsbHLH062 plays a redundant role with OsbHLH063 in regulating Fe homeostasis under Fe-sufficient conditions. Furthermore, the unaltered Fe concentrations in rice plants lacking *OsbHLH062* function may be attributed to the critical role of OsbHLH061 in preventing shoot Fe accumulation, potentially masking the function of OsbHLH062. Future studies should aim to clarify these hypotheses using double and triple mutants involving *OsbHLH061*, *OsbHLH062*, and *OsIRO3*. Overexpression lines are a valuable approach for exploring the functions of genes with redundant roles, and the results from *OsbHLH062* overexpression lines indicated that OsbHLH062 negatively regulates Fe-deficiency responses. *OsbHLH062*-overexpressing plants were more sensitive to Fe deficiency than the WT, as demonstrated by significantly lower SPAD values of new leaves and lower shoot Fe concentrations in response to Fe deficiency (Fig. 3). In addition, the induction of Fe-deficiency-responsive genes was suppressed in the *OsbHLH062* overexpression lines (Fig. 5).

The different phenotypes observed among the *osbhlh061*, *osbhlh062*, and *osiro3* single mutants can be explained by their expression patterns. For instance, *OsbHLH061* is repressed by Fe deficiency and primarily functions under Fe-sufficient conditions (Wang et al. 2022b), whereas *OsIRO3* is induced by Fe deficiency and mainly operates under Fe-deficient conditions (Wang et al. 2020c). Our RT-qPCR results indicated that *OsbHLH062* is induced in the roots but repressed in the shoots in response to Fe deficiency (Fig. 2). The contrasting responses of *OsbHLH062* expression to Fe deficiency suggest that some genes are regulated by OsbHLH062 to different extents in the root and the shoot. Although OsbHLH062 and OsIRO3 are closely related evolutionarily and share many functional similarities, the new leaves of *osbhlh062* mutants did not exhibit necrotic spots, unlike those of *iro3* mutants (Carey-Fung et al. 2022; Li et al. 2022a; Wang et al. 2020a, 2020c).

In *Arabidopsis*, studies of mutants with loss-of-function mutations of PYE (an ortholog of OsIRO3) and IAA LEUCINE RESISTANT 3 (ILR3) revealed that defects in photosystem II (PSII) lead to production of reactive oxygen species (ROS) under Fe-deficient conditions (Akmakjian et al. 2021). Therefore, we propose that ROS production in the *iro3* mutants may also result from defects in PSII. Our GUS staining results in *OsbHLH062*_*Pro*_*:GUS* transgenic plants showed that *OsbHLH062* was minimally expressed in mesophyll cells (Fig. 2E), where chloroplasts are located. Thus, OsbHLH062 is unlikely to play a role in photoprotection in response to Fe deficiency. The functional divergence between OsIRO3 and OsbHLH062 may be due to differences in their expression patterns. Recent studies have shown that the mRNA expression patterns governed by the promoters of two bHLH TFs, phytochrome-interacting factors 1 and 4 (PIF1 and PIF4), play an important role in the functional diversification of these proteins (Kim et al. 2024).

Generally, bHLH transcription factors regulate downstream target genes by forming homodimers or heterodimers (Li et al. 2006). We confirmed that OsPRI1, a master positive regulator of *OsIRO2* expression, interacts with OsbHLH062, forming a OsbHLH062–OsPRI1 bHLH heterodimer (Fig. 6A and B). Furthermore, we demonstrated the repression of OsPRI1 transactivation by OsbHLH062 using a dual LUC reporter assay (Fig. 6C). Notably, *OsIRO2* expression was upregulated in the *osbhlh062* mutants under Fe-sufficient conditions, but this upregulation was not seen in *osbhlh062* mutants in the *pri1* mutant background (Fig. S6 and Fig. 6D–F). This suggests that the OsbHLH062–OsPRI1 heterodimer acts upstream of *OsIRO2*, similar to the interactions between OsbHLH061–OsPRI1 and OsIRO3–OsPRI1 (Li et al. 2022a; Wang et al. 2022c).

Overall, this work identified a previously uncharacterized bHLH regulatory module that regulates the expression of *OsIRO2*, a key gene required for Fe uptake. Similarly, several bHLH heterodimers have been discovered in *Arabidopsis*. Arabidopsis bHLH Ib subgroup TFs (AtbHLH38, AtbHLH39, AtbHLH100, and AtbHLH101) are orthologs of OsIRO2. These bHLH Ib subgroup members interact with FIT to regulate the expression of genes involved in Fe uptake via Strategy I (Colangelo and Guerinot 2004; Wang et al. 2013; Yuan et al. 2005, 2008). The expression of bHLH Ib genes is directly regulated by dimers of bHLH IVc TFs. Additionally, the bHLH IVb proteins bHLH121, PYE, and bHLH11, interact with ILR3 to form heterodimers that function upstream of genes involved in regulating Fe homeostasis (Gao et al. 2020, 2021; Kim et al. 2019; Lei et al. 2020; Li et al. 2022b; Tissot et al. 2019; Zhang et al. 2015). We propose that the regulation of Fe homeostasis by bHLH dimers is conserved throughout plant species.

In summary, we identified a previously uncharacterized bHLH TF, OsbHLH062, which plays an important role in maintaining Fe homeostasis. Similar to the bHLH TFs OsbHLH061 and OsIRO3, OsbHLH062 contains an EAR motif in its C-terminal domain, and this motif is required for its transcriptional repression activity and the recruitment of OsTPL/TPR co-repressors. We demonstrated that OsbHLH062 functions as a transcriptional repressor that negatively regulates Fe-deficiency responses in rice. The results from this study provide valuable insights that will facilitate the development of Fe-biofortified rice plants, helping us to tackle the global challenge of Fe deficiency.

## Materials and methods

### Plant materials and growth conditions

Rice (*Oryza sativa*) seedlings were grown in a greenhouse with a photoperiod of 14 h of light at 30 °C and 10 h of darkness at 25 °C. For hydroponic experiments, seeds were germinated in water at 37 °C for 2 days and then transferred to a 0.5 mM CaCl_2_ solution in darkness for 3 d, followed by normal light conditions for 1 day. Seedlings were then cultured in half-strength Kimura B solution, which contained 0.18 mM (NH_4_)_2_SO_4_, 0.27 mM MgSO_4_, 0.09 mM KNO_3_, 0.18 mM Ca(NO_3_)_2_, 0.09 mM KH_2_PO_4_ (pH 5.5), 0.50 μM MnCl_2_, 3.00 μM H_3_BO_3_, 1.00 μM (NH_4_)_6_Mo_7_O_24_, 0.40 μM ZnSO_4_, 0.20 CuSO_4_, and 2.00 μM FeSO_4_. The nutrient solution was renewed every 2 d, and the pH was adjusted to pH 5.50 each time. For Fe-deficiency treatments, no external Fe was added to the solution. For excess Fe treatment, the solution contained 200 μM FeSO_4_.

### Production of genetically altered plant materials

Single mutants of *OsbHLH062* were generated using CRISPR/Cas9 technology (Xie and Yang 2013). Two independent guide RNAs (gRNAs) targeting exon 1 of *OsbHLH062* were selected from the CRISPR-PLANT database (http://omap.org/crispr/CRISPRsearch.html) for gene editing and ligated into the vector pRGEB32. For construction of the double mutant vector, the gRNA 1 of *OsbHLH062* or the gRNA of *OsPRI1* (Wang et al. 2022b) was inserted into the SK-gRNA vector cut with *Aar*I. The resulting vectors were separately digested with *Xba*I/*Bgl*II and *Kpn*I/*Nhe*I, and the fragments with gRNA were ligated into the pC1300-Cas9 vector as previously reported (Wang et al. 2015). Homozygous mutants were identified through polymerase chain reaction (PCR) and Sanger sequencing. Frameshift mutations were detected in the resulting T2 and T3 generations of two independent homozygous mutants of *OsbHLH062* (*osbhlh062-1* and *osbhlh062-2*) and double mutants of *OsbHLH062* and *OsPRI1* (*osbhlh062 pri1-1* and *osbhlh062pri1-2*). Detailed information regarding target sites, gene editing, and the predicted mutant proteins is shown in Figs. S2 and S10.

To generate *OsbHLH062* overexpression lines, the *OsbHLH062* coding sequence (CDS) was amplified from ‘Nipponbare’ cDNA and then inserted into pGWB2 using the Gateway technique (Nakagawa et al. 2007). To generate *OsbHLH062*_*pro*_*:GUS* transgenic plants, a 2,137-bp promoter sequence from *OsbHLH062* was cloned into a modified pBI101.3 binary vector by homologous recombination using a ClonExpress® II One Step Cloning Kit (Vazyme, Nanjing, China), placing it in frame with GUS-Plus to generate the vector *OsbHLH062*_*Pro*_:*GUS*. The newly constructed vectors were transformed into rice calli via *Agrobacterium*-mediated genetic transformation (Chen et al. 2003). Sequences of the primers used for vector construction and amplification of the target genes are given in Table S1.

### Subcellular localization analysis

To examine subcellular localization of OsbHLH062, the CDS without stop codon of OsbHLH062 was cloned into the N terminus of GFP in the pYL322-d1-eGFP vector to generate *CaMV35S*_*pro*_*:OsbHLH062-GFP* vector. The *CaMV35S*_*pro*_*:OsAPI5-mCherry* vector was used as a nuclear marker. The co-transformed OsbHLH062-GFP and API5-mCherry fusion proteins were transiently expressed in rice protoplasts, as described previously (Wang 2022a). As a negative control, *CaMV35S*_*pro*_:GFP and *CaMV35S*_*pro*_*:OsAPI5-mCherry* vectors were also co-transformed. The fluorescence signals were observed using a laser confocal microscope.

### Transcription activity analysis

For analysis of transcription activity, the *GAL4*_*pro*_:*LUC* reporter construct and the effectors *35S:BD-OsbHLH061* and *35S:BD-OsbHLH061*^EAR L−A^ (*35S:BD-OsbHLH061DM2*) were used as described previously (Wang et al. 2022b). To construct *35S:BD-OsbHLH062* and *35S:BD-OsbHLH062*^EAR L−A^ effector vectors, sequences encoding OsbHLH062 and the mutated EAR motif were fused with those encoding the BD domain driven by the 35S promoter in the modified plasmid pCAMBIA1300-BD using a ClonExpress II One Step Cloning Kit (Vazyme, Nanjing, China). The mutated sequence of OsbHLH062 was synthesized by MiniGene™ synthesis by Azenta (Suzhou, China). The reporter construct *GAL4*_*pro*_:*LUC* was introduced into *Agrobacterium* strain EHA105 together with p19 constructs (Hellens et al. 2005), and the effectors or the empty vector were introduced into EHA105 alone.

For transformation of *Nicotiana benthamiana* leaves, the above agrobacteria were cultured, harvested, and resuspended in Murashige and Skoog-MES medium containing 150 μM acetosyringone (pH 5.6), according to a previous study (Chen et al. 2008). The bacterial cultures were then mixed and infiltrated into *N. benthamiana* leaves. The infiltrated leaves were sampled for measurement of LUC and REN activities using a Dual-Luciferase Reporter Assay Kit (Yeasen, Shanghai, China). The ratio of LUC activity to REN activity under control conditions was set to 1. Sequences of the primers used for transcription activation assays are listed in Table S1.

### Yeast two-hybrid assay

The matchmaker GAL4 Y2H system (Clontech) was used to detect the interaction of OsbHLH062 with OsTPL/TPR proteins or OsPRI1/2/3/4. The CDSs of *OsbHLH062* and *OsbHLH062 *^EAR L−A^ were cloned into *Eco*RI + *Bam*HI-digested pGBKT7 (Clontech) by homologous recombination using a ClonExpress^®^ II One Step Cloning Kit (Vazyme, Nanjing, China). *BD-OsTPLN*, *BD-OsTPR1N*, and *BD-OsTPR2N*, which were derived from OsTPL/TPR proteins fused with BD in pGBKT7 (Clontech), were used as described previously (Wang et al. 2022b). For construction of *AD-OsbHLH062* and *AD-OsPRI1/2/3/4*, the full-length CDSs of *OsbHLH062* and *AD-OsPRI1*, respectively, were fused with AD sequences in pGADT7 (Clontech). Different combinations of the final constructs were transformed into AH109 yeast (*Saccharomyces cerevisiae*) cells. The transformed cells were spotted onto synthetic dropout nutrient medium lacking tryptophan and leucine (SD/–Trp/–Leu) and synthetic dropout nutrient medium lacking tryptophan, leucine, histidine, and adenine (SD/–Trp/–Leu/–His/–Ade) to test for interactions. The growth of yeast cells was evaluated after incubation at 30 °C for 2 to 3 days.

### Split-LUC complementation assay

The CDS of *OsbHLH062* lacking the stop codon was amplified from rice cDNA and inserted into pCAMBIA1300-nLUC (Chen et al. 2008) by recombination reactions using a ClonExpress^®^ II One Step Cloning Kit (Vazyme, Nanjing, China). Sequences encoding OsTPL and OsPRI1/2/3/4 were fused with those encoding cLUC in pCAMBIA1300-cLUC (Chen et al. 2008) to generate cLUC-OsTPL and cLUC-OsPRI1, which have been used previously (Wang et al. 2022b). GFP fused with cLUC or nLUC to generate GFP-nLUC or cLUC-GFP served as a negative control. To confirm the interaction of OsbHLH062 with OsTPL or OsPRI1, OsbHLH062-nLUC with either cLUC-OsTPL or cLUC-OsPRI1 was co-transformed into leaf cells of *N. benthamiana* via *Agrobacterium*-mediated transformation. In addition, the combinations cLUC-TPL and OsMYC2-nLUC, cLUC-GFP and OsbHLH062-nLUC, cLUC-GFP and OsMYC2-nLUC, cLUC-OsPRI1/2/3/4 and GFP-nLUC, and cLUC-TPL and GFP-nLUC, were used as negative controls.

For *Agrobacterium*-mediated transformation, *Agrobacterium* strain EHA105 containing the appropriate vector(s) was incubated at 30 °C for 36 h; 1 mL of the above culture was then transferred into 20 mL *Agrobacterium rhizogenes* medium and incubated for 6 h at 30 °C. The bacteria were resuspended in Murashige and Skoog-MES medium (pH 5.6) containing 150 μM acetosyringone to a final concentration of OD_600_ = 0.5. Finally, equal volumes of the different *Agrobacterium* strains were mixed and co-infiltrated into *N. benthamiana* leaves using a 1-mL disposable syringe (Chen et al. 2008). Transformed *N. benthamiana* plants were grown in darkness for 12 h, followed by 36 h of growth under normal light conditions (16-h light, 26 °C; 8-h dark, 22 °C). LUC signals were examined using a Tanon 5200 Multi automatic chemiluminescence/fluorescence image analyzer (Tanon, Shanghai, China). Sequences of the primers used for construction of vectors are given in Table S1.

### Pull-down assay

Sequences encoding the N-terminus of OsTPL or OsPRI1 fused with those encoding GST in the pGEX-4 T-1 vector. The full-length CDS of *OsbHLH062* was cloned into the pET-32a (+) vector to fuse with His. The resulting vectors were transformed into *Escherichia coli* BL21 (DE3) cells. GST-OsTPLN, GST-OsPRI1, and GST were expressed in BL21 cells grown in Luria–Bertani medium that were induced with 0.1 mM isopropyl-β-thiogalactopyranoside (IPTG) at 16 °C for 20 h; these GST fusion proteins were purified from *Escherichia coli* BL21 cells using a MagneGST™ Glutathione Purification System (Promega, USA) according to the manufacturer’s recommendations. Expression of the His-OsbHLH062 fusion protein in BL21 cells was induced by 0.1 mM IPTG at 16 °C for 20 h, and the protein was purified using a Ni-IDA-Sefinose™ Resin Kit (Sangon, China) following the manufacturer’s protocol after lysis of cells by ultrasound. For pull-down assays, the purified His-OsbHLH062 fusion protein was incubated with immobilized GST, nd GST-OsTPLN, or GST-OsPRI1 at 4 °C for 2 h. The beads were washed five times with ice-cold MagneGST™ Binding/Wash Buffer (pH 7.4), and proteins were released from the beads by boiling in SDS sample buffer for 5 min, followed by detection via immunoblot analysis. For immunoblotting, anti-His conjugated to horseradish peroxidase (Santa Cruz, sc-8036 HRP) and anti-GST conjugated to horseradish peroxidase (Santa Cruz, sc-138 HRP) were used at 1:5000 dilutions. Sequences of the primers used for pull-down assays are listed in Table S1.

### Histochemical GUS staining

GUS staining was performed using *OsbHLH062*_*Pro*_:*GUS* and transgenic plants as follows. Roots and leaves of seedlings grown in nutrient solution containing 0 or 2 μM FeSO_4_ were harvested and used for histochemical GUS staining as described previously (Wang et al. 2020c). Samples were infiltrated with staining solution in a vacuum for 30 min and incubated at 37 °C overnight. Roots and leaves were then photographed using a ZEISS Stemi 2000C stereomicroscope (ZEISS, Germany). A vibratome (VT1200S, Leica, Nussloch, Germany) was used to prepare root and leaf Sects. (20- to 100-μm thickness), which were then photographed under a microscope (DM500, Leica, Nussloch, Germany).

### Phenotype analysis and measurement of Fe concentrations

To investigate the performance of *OsbHLH062* mutants and overexpression lines in response to Fe deficiency, 14-day-old wild-type and transgenic seedlings were transferred to nutrient solutions supplemented with 2 or 0 μM FeSO_4_. After 8 days, the SPAD values of the newly expanded leaves were measured using a portable Soil and Plant Analysis Development chlorophyll meter (SPAD-502; Minolta Sensing) and the growth of the plants was recorded using a camera. Root and shoot lengths were measured, and samples were collected for determining Fe concentrations. For the excess Fe treatment, 14-day-old WT, *osbhlh061*, and *osbhlh062* mutant plants were transferred to a solution containing 2 or 200 μM FeSO_4_ and cultured for another 15 days.

Measurement of Fe concentrations in roots and shoots was performed as described previously (Dong et al. 2018). Briefly, samples were dried at 80 °C for 3 days and then digested in 3 mL of HNO_3_/HClO_4_ (87:13, v/v) in an electrothermal digestion apparatus (DigiBlock ED54; LabTech, Hopkinton, MA, USA). Fe concentrations were measured using an inductively coupled plasma mass spectrometer (ICP-MS; NexION 300X; Perkin-Elmer, Waltham, MA, USA).

### RNA isolation and RT-qPCR

Total RNA was extracted from rice roots using a FastPure^®^ Plant Total RNA Isolation Kit (Vazyme, Nanjing, China) following the manufacturer’s instructions. First-strand cDNAs were synthesized from total RNA using a HiScript® III SuperMix for qPCR Kit with gDNA wiper (Vazyme, Nanjing, China). RT-qPCR was performed using ChamQ^™^ SYBR^®^ Color qPCR Master Mix (Vazyme, Nanjing, China) on a Mastercycler^®^ EP realplex real-time PCR system (Eppendorf, Hamburg, Germany). The relative expression level of genes was calculated using the Eq. 2^–∆∆Ct^, with *OsActin1* as an internal reference. Sequences of the primers used for qRT-PCR are listed in Supplemental Table S1. All experiments were performed with three biological replicates.

### Accession numbers

Sequence data of genes in this study can be found in the Rice Genome Annotation Project database as follows: *OsbHLH061* (LOC_Os11g38870), *OsbHLH062* (LOC_Os07g43530), *OsIRO3* (LOC_Os03g26210), *OsIRO2* (LOC_Os01g72370), *OsNAS1* (LOC_Os03g19427), *OsNAS2* (LOC_Os03g19420), *OsNAAT1* (LOC_Os02g20360), *OsTOM1* (LOC_Os11g04020), *OsYSL15* (LOC_Os02g43410), *OsIRT1* (LOC_Os03g46470), *OsIRT2* (LOC_Os03g46454), *OsDMAS1* (LOC_Os03g13390), *OsTPL* (LOC_Os03g14980), *OsTPR1* (LOC_Os08g06480), *OsTPR2* (LOC_Os01g15020).

## Data Availability

All data supporting the findings of this study are available within the paper and its Supplementary Information.
